# Skull defect increases the tumor treating fields strength without detrimental thermogenic effect: A computational simulating research

**DOI:** 10.1002/cam4.5037

**Published:** 2022-07-21

**Authors:** Taian Jin, Zhangqi Dou, Yu Zhao, Biao Jiang, Jinghong Xu, Buyi Zhang, Boxing Wei, Fei Dong, Jianmin Zhang, Chongran Sun

**Affiliations:** ^1^ Department of Neurosurgery, The Second Affiliated Hospital Zhejiang University School of Medicine Hangzhou Zhejiang China; ^2^ Jiangsu Hailai Xinchuang Medical Technology Co., Ltd. Wuxi Jiangsu China; ^3^ Department of Radiology, The Second Affiliated Hospital Zhejiang University School of Medicine Hangzhou Zhejiang China; ^4^ Department of Pathology, The Second Affiliated Hospital Zhejiang University School of Medicine Hangzhou Zhejiang China; ^5^ Key Laboratory of Precise Treatment and Clinical Translational Research of Neurological Diseases Hangzhou Zhejiang China; ^6^ Clinical Research Center for Neurological Diseases of Zhejiang Province Hangzhou China

**Keywords:** finite element analysis, glioblastoma, skull defect, tumor treating fields

## Abstract

**Background:**

Tumor treating fields (TTFields) is an FDA‐approved adjuvant therapy for glioblastoma. The distribution of an applied electric field has been shown to be governed by distinct tissue structures and electrical conductivity. Of all the tissues the skull plays a significant role in modifying the distribution of the electric field due to its large impedance. In this study, we studied how remodeling of the skull would affect the therapeutic outcome of TTFields, using a computational approach.

**Methods:**

Head models were created from the head template ICBM152 and five realistic head models. The electric field distribution was simulated using the default TTFields array layout. To study the impact of the skull on the electric field, we compared three cases, namely, intact skull, defective skull, and insulating process, wherein a thin electrical insulating layer was added between the transducer and the hydrogel. The electric field strength and heating power were calculated using the FEM (finite element method).

**Results:**

Removing the skull flap increased the average field strength at the tumor site, without increasing the field strength of “brain”. The ATVs of the supratentorial tumors were enhanced significantly. Meanwhile, the heating power of the gels increased, especially those overlapping the skull defect site. Insulation lightly decreased the electric field strength and significantly decreased the heating power in deep tumor models.

**Conclusion:**

Our simulation results showed that a skull defect was beneficial for superficial tumors but had an adverse effect on deep tumors. Skull removal should be considered as an optional approach in future TTFields therapy to enhance its efficacy. An insulation process could be used as a joint option to reduce the thermogenic effect of skull defect. If excessive increase in heating power is observed in certain patients, insulating material could be used to mitigate overheating without sacrificing the therapeutic effect of TTFields.

## Graphical abstract

Of all the tissues the skull plays a significant role in modifying the distribution of the electric field due to its large impedance. In this study, influence of remodeling of the skull on the therapeutic outcome of TTFields have been studied.

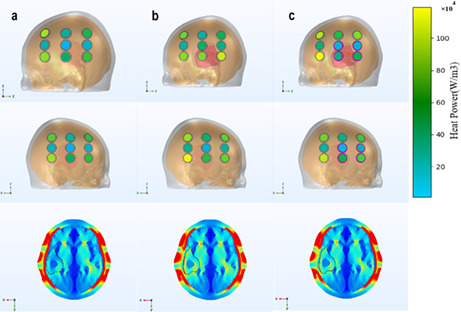

## 1 INTRODUCTION

Glioblastoma (GBM), which has a global estimated incidence of nearly 3.4 per 100,000 people, is the most aggressive primary malignant brain tumor and has a grim prognosis.^1^ Even with maximal safe resection, followed by chemotherapy such as temozolomide (TMZ), with concurrent and adjuvant radiotherapy, the median overall survival (OS) is only 14.6 months.^2^ Therefore, there is an urgent need to develop powerful therapeutic approaches for this devastating disease.

Tumor‐treating fields (TTFields) is an FDA‐approved novel treatment for both newly diagnosed and recurrent GBM, based on two positive clinical trials (EF‐11 and EF‐14) which demonstrated the efficacy and safety of TTFields in prolonging progression‐free survival and OS when combined with TMZ.^3^, ^4^, ^5^ TTFields locoregionally delivers low‐strength alternating electric fields (200 kHz) to the tumor via external transducer arrays placed on the scalp, and have been recognized as a non‐invasive antimitotic cancer treatment.^6^ According to previous in vitro and in vivo investigations, TTFields can inhibit cell proliferation, disrupt cell division, interfere with cell migration and invasion, and reduce DNA repair.^7^, ^8^, ^9^ Albeit with the encouraging therapeutic effects of TTFields, more detailed evidence regarding the correlation between the profile of the electric field strength and the clinical outcome is still under investigation.^9^, ^10^ More importantly, the thermogenic effect, i.e., heat production of the scalp‐affixed arrays, is believed to be associated with the most common adverse events, namely skin irritation, such as rash, and heat sensation.^5^, ^11^ The proper regulation of heat production during TTFields usage is conducive to relieving the skin reaction.

A computational approach, such as finite element method (FEM), is commonly used to explore the impacts of different tissue structures e.g., white matter and tumor shell, distinct tissue conductivities, and layout of transducer arrays on electric field distribution within the brain.^6^, ^12^ Moreover, FEM studies can optimize TTFields treatment planning for individual cases, such as recurrent GBM and posterior fossa lesions.^10^, ^13^ Of all the tissues, the skull plays a significant role in modifying the distribution of the electric field with its large impedance. For patients with intact skulls, the distribution of the electric field can be estimated with credible accuracy for two reasons. First, the flexibility of the transducer array layout is largely limited by the size and topological structure of the skull. Second, the scalp temperature is monitored in real‐time and the applied voltage is accordingly adjusted to avoid overheating; in other words, a constant voltage is maintained when the heat transfer process reaches balance.^14^ Although surgical treatment does not require removal of a bone flap for most patients diagnosed with GBM, decompressive craniectomy is performed in cases with high intracranial pressure. Under these circumstances, the redistribution of the electric field due to the skull defect remains poorly understood. It is hypothesized that removal of a specific bone flap can increase the electric field strength in the brain. A phase I trial has proven the safety of the combination of TTFields and skull‐remodeling surgery; moreover, a phase II trial was initiated to test the efficacy of skull remodeling surgery.^15^, ^16^ However, the amplified electric field is accompanied by increased heat production. Therefore, computational modeling of the distribution of the electric field and thermogenic effects in such patients is important, because overheating in patients with skull defects will force the application of a lower voltage, which might decrease the focal electric field in the tumor.^14^, ^17^

In this study, we applied the FEM approach to ascertain the electric field distribution inside GBM head models, with or without skull defects, and the impact of an insulation layer thereon. This may provide new insights into the application scope of TTFields, and the strategy of utilizing skull defects to maintain field strength, avoid overheating, and maximize antineoplastic efficiency.

## 2 MATERIALS AND METHODS

### 2.1 Patient selection and MRI acquisition

Each patient's images were acquired through the local institutional review board review committee and informed consent was obtained from the patients. Patients with a new pathologic diagnosis of glioblastoma were included. Moreover, images were reviewed to ensure the selected tumors were in a single lobe or brainstem and harboring a significant central necrotic area. Necrotic areas, as the tumor cores, are necessary for analysis in FEM. Cranial MRI was acquired with the same 3T scanner. Radiological features were extracted from axial T1‐W, T2‐W and enhanced T2‐W images. The standard brain template ICBM152 image (International Consortium for Brain Mapping) was downloaded from the website http://nist.mni.mcgill.ca/.

### 2.2 Creating the head models

The head template, ICBM152, and five realistic head models (the location of the tumor was respectively unilateral frontal, temporal, insular, and parietal lobe, and brainstem) created from MRI data were used to construct five head models with embedded tumors. After aligning the images to MNI (Montreal Neurological Institute) space, the coordinates of real head models were unified with the ICBM152.^18^ The ICBM152 image was segmented into scalp, skull, cerebrospinal fluid (CSF), gray matter (GM), white matter (WM) and air. Additionally, the five virtual tumors were manually separated from the images and segmented into the tumor shells and tumor cores. After restoration with Geomagic studio software, the tissues were imported into COMSOL Multiphysics to generate geometry models. Then the geometry model of each tissue was combined to form the complete head model. Each tissue was finely meshed after the geometry files were generated, and then the grid coordinates of each mesh were derived. A one‐to‐one mapping between the grid coordinates of each tissue and its electrical properties was made (refer to https://itis.swiss/virtual‐population/tissue‐properties/database/dielectric‐properties/ Table 1). To simplify the study, we did not include the field correlation and anisotropic conductivity which may affect the intracranial electric field distribution.^19^, ^20^ Moreover, the electrical properties of the head composition can be obtained by constructing an interpolation function for the mapping relationship between all tissue coordinates of the entire head and the corresponding electrical properties. After assigning electrical properties to the head geometry model, the corresponding electric field distribution can be calculated on this model.

**TABLE 1:** Electrical parameters required as inputs for computer modeling and calculation.

	Relative permittivity, *ε*	Elec. Cond., *σ* (S/m)
Scalp	5000	0.25
Skull	204	0.0211
Gray matter	2010	0.141
White matter	1290	0.0868
Cerebrospinal fluid	109	2.00
Tumor shell	2000	0.24
Tumor core	110	1.00
Hydrogel	100	0.1
Electrode	16,000	0
Air	1	0

### 2.3 Creating the head models with tumors

The results generated by segmentation were all exported as NIFTI files. Then each tumor and the ICBM152 tissues were imported into the 3D slicer software and the “subtract” operation was run to solve tissue overlap issues. After five operations, the tumors were implanted into the template, that is, five head models with tumors were obtained.

### 2.4 Creating the head models with skull defects

Based on the location of the tumor, the position and size of the skull defect were determined by a senior neurosurgeon. Considering that the defective part of the skull would be filled with scalp tissue, the electrical properties of this part of the tissue were consistent with the scalp in the calculations of the skull defect models. Finally, the interpolation function of the tissue coordinates and the electrical properties of a head with a skull defect was established and assigned to the head model, that is, head models with a skull defect were obtained.

### 2.5 Placing the electrode arrays

Usually, two pairs of arrays are attached to the patient's head, i.e., left–right arrays (LR arrays) and anterior–posterior arrays (AP arrays). In this study, to compare the effect of the skull defect on the electric field, only one pair of electric arrays was added, depending on the location of the skull defect. Each array consisted of a total of 3 × 3 transducers, which were electrode cylinders with a height of 1.0 mm and a radius of 10 mm. Based on the default layout of the electrode arrays (symmetric configuration) and the relative position of the transducers in the electrode arrays, the electrode sheets were added to the previously established head geometry model.^21^ Meanwhile, the thin layer between the electrode and the scalp with 1.0 mm thickness and 12.5 mm radius represented the hydrogel that adheres the electric arrays to the scalp.

### 2.6 Calculating the electric field strength and heating power

The electric field distribution within the brain was calculated using the FEM to solve the electro quasistatic approximation of the Maxwell equations, which are valid for these models.^22^ So, after modeling the complete head with the electrode sheets and the gel, we performed FEM using COMSOL Multiphysics software. One side of the electrode sheet was grounded and 80 V was applied to the other side. Current was controlled at the array level but not at the transducer level, so the transducers would work at the same time. Zero normal component of the current density (electric insulation) was applied to the rest of the outer boundary. The electric field can be calculated according to Formula 1. Concurrently, the Joule heating function (Formula 2) was calculated to represent tissue overheating due to long‐term exposure to TTFields.

(1) $$\nabla ∙\left(\left(\sigma +\mathrm{i\omega \varepsilon}\right)\;E\right)=0$$

where σ (S/m) is the electrical conductivity of tissues, ω is the angular frequency, ε is the relative permittivity, and E (V/m) is the electric field strength.

(2) Qrh=12ReJ∙E*

where Q (W/m^3^) is the heating power, J (A/m^2^) is the current density, and $E^{*}$ is the conjugate of electric field strength.

### 2.7 Placing the electric insulating layer

Considering the strong electric field strength at the defect site, there would be a significant increase in thermogenesis. To control the thermogenic effect, a scheme in this study, adding a thin electric insulating layer to the contact surface between the transducer and the hydrogel, was proposed. The electric insulating layer, with a thickness of 1 μm, a dielectric constant of 20 mm, a relative permittivity of 2.2, negligible conductivity, a specific heat capacity of 2.3 J/(kg·°C) and a heat conductivity of 0.42 W/m·K, was deposited on the inner surface of transducers at the site of the skull defect. Since the thickness of the insulating layer was negligible compared to that of the transducer, the boundary condition of contact impedance was applied to simplify the calculation.

### 2.8 Statistical analysis

The distribution of the intracranial electric field and the heating power of the gel were calculated according to above formulas, and were then exported and processed as colorful diagrams with color scales. Other data, including the average electric field strength and above‐threshold volumes (ATVs) of each tissue, were calculated in the COMSOL software and exported as needed.

## 3 RESULTS

### 3.1 Demographics

Patients with GBM and their imaging data were used in this study. The five included patients were 18–63 years old; their tumors were confined to the unilateral frontal, insular, temporal, and parietal lobes, and the brainstem, respectively; and the tumor volumes ranged from 22.36 to 114.28 cm^3^. The ratio of males to females was 3:2.

### 3.2 Electric field distribution within the brain

**FIGURE 1:**
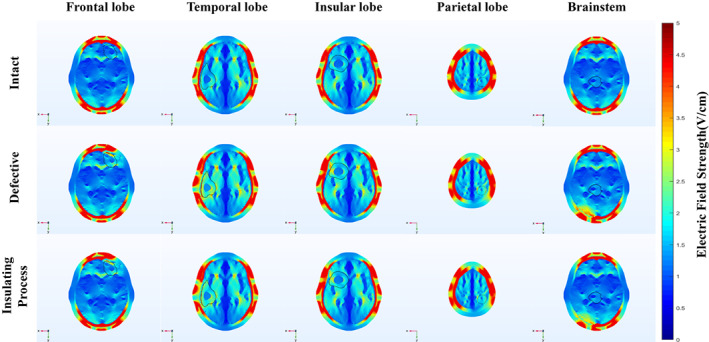
The electric field distribution in the tumor plane for each model, with the black circled part being the tumor. The intracranial field strength of the LR arrays (temporal, insular, and parietal lobe models) was stronger than that of the AP arrays (frontal lobe and brainstem models). The maximum field strength appeared in the scalp and skull near the transducers, and the minimum field strength appeared at the midline of the connecting line of both arrays. After removing the bone flap, obvious field strength enhancement was visible at the tumor shell. The expansion of the “hot spot” scope can be observed when comparing the intact skull and the defective skull cases. The electric field strength of the tumor shells appeared to be significantly enhanced, but that of the tumor core remained almost unchanged. After the insulating process, the field strength and volume of the “hot spot” of each model were similar to that before processing.

Depending on the tumor location, the electric field distribution was calculated only for a single pair of arrays, i.e., LR or AP arrays. Because the location of the skull defect and transducers were fixed, the skull defect only affected the pair of arrays with which there was a contact. The intracranial field distribution in the horizontal plane of each tumor is shown in Figure 1 by simulating the five models using FEM. Figure 1 shows that, in all models, the distribution of the intracranial field was not uniform due to the effect of complex tissue interface shapes and differences in the electrical conductivity of different tissues. Overall, although the field distribution within the brain was still symmetric in the presence of a tumor, the intracranial field strength of LR arrays (temporal, insular, and parietal lobe models) was significantly larger than that of AP arrays (frontal lobe and brainstem models). The field strength of tissues near the transducer location was larger, the maximum field strength was visible in the scalp and skull underneath the transducers, and the field strength of tissues far from the transducer location was smaller.

In addition, a “hot spot” formed at the interface of high and low conductivity tissues, which were not single points but regions with local field enhancement, as the field strength increased in the low conductivity tissues and decreased in the high conductivity tissues. In comparison, after bone flap removal, all models' tumor shell showed the expansion of volume and scope of “hot spot” activity except the brainstem model. Moreover, there was no significant decrease in hot spot scope and field strength after adding the insulating layer.

**FIGURE 2:**
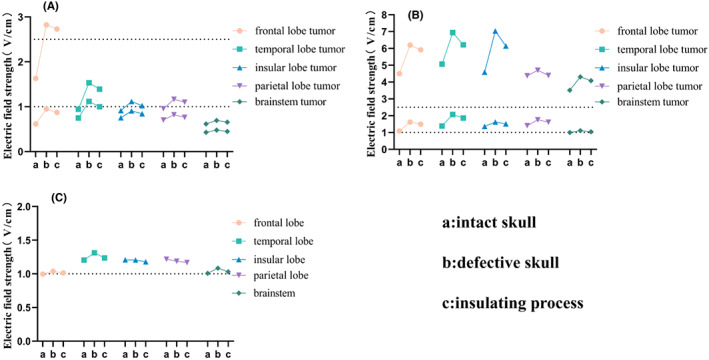
Average and maximum field strength of the tumor core (A), tumor shell (B) and “brain” (including GM, WM and CSF) (C) for each model; the upper line was the maximum value for each case in each model and the lower line was the average value for each case in each model. The average field strength of the shells was significantly larger than that of the cores due to the low conductivity of the shells. Moreover, the tumor shell parts were more sensitive than the tumor core parts. The electric field strength of “brain” changes little when comparing the three different cases. For all models, the average electric field strength of all the tumor shells and the “brain” exceeded the treatment threshold of 1 V/cm.

**FIGURE 3:**
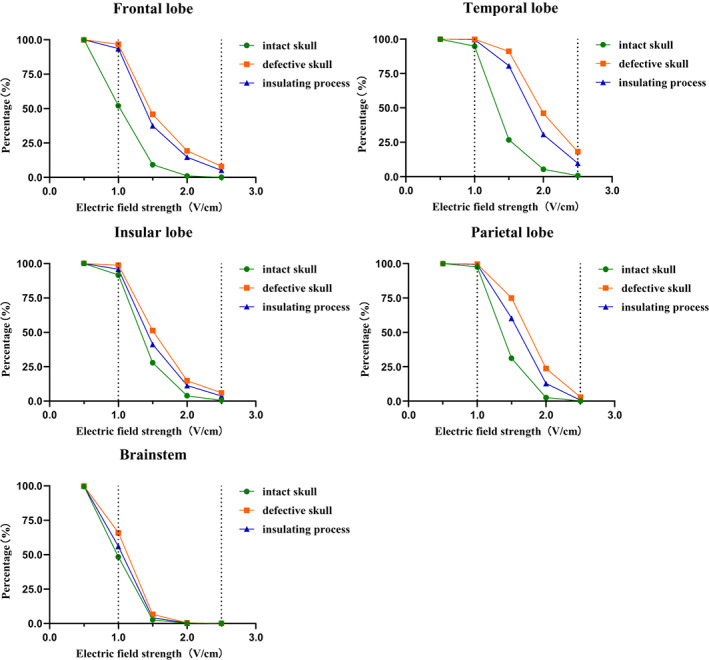
Above‐threshold volumes (ATVs) for each model, representing the tumor shell's volume with a field strength above a certain value. The ATVs in the skull defect models were greater than that in the insulating process models, whose ATVs were greater than in the intact skull models. ATVs in models with LR arrays (temporal, insular, and parietal lobes) were more sensitive than that with AP arrays (frontal lobe and brainstem). Models with deep tumors (insular lobe and brainstem) possessed less ATVs when the skull was intact, but there was a large improvement in ATVs in the insular lobe model after removing the skull flap.

In addition, at the tumor site, our region of interest, the field strengths of the tumor core, tumor shell and “brain” (including WM, GM, CSF) were calculated, and the results are shown in Figures 2 and 3. Figure 2 shows the effects of different processes on the electric field of the tumor. As “brain” accounted for most of the intracranial volume, skull defects had little impact on it. Both the average field strengths of the shell and “brain” of each model were greater than 1.0 V/cm, which reached the threshold value for inhibition of tumor proliferation. The average field strength of the “brain” of the models which shared the same arrays were similar.

Comparing each case in the same model, Figure 3 showed that the ATVs in the skull defect cases were greater than that in the insulation process cases, which in turn was greater than that of the intact skull cases. However, in the brainstem model, the difference in ATVs between the cases was not significant. Comparing the different models, in the intact skull case, the ATV 1.0 was only about 50% in the frontal lobe and brainstem models, but greater than 90% in the temporal, parietal and insular lobe models, indicating that the overall field strengths were smaller in the AP arrays compared to the LR arrays. After bone flap removal, the ATVs increased in all models, with larger increases in the frontal, temporal, and parietal lobe models and smaller increases in the insular lobe and brainstem models, which was considered to be related to the depth of the tumor.

### 3.3 The thermogenesis of TTFields in each model

Although some encouraging results can be seen, we can increase the field strength to the tumor shell, as well as the intracranial field strength to some extent, by removing the bone flap. However, this created another problem i.e., the skin dermatitis, which is the main side effect of the electric field treatment, was caused by overheating of the electrode sheet and gel. While removing part of the skull increased the overall field strength due to the lack of the voltage dividing effect of the skull, the heating power of the gel increased. Concurrently, the cost of the temperature increase was reduction of voltage to maintain an equilibrium between heat production and dissipation, with the direct consequence that the increase in field strength was not sustainable, and the final intracranial field strength at equilibrium needed to be further inferred and calculated based on the heating power.

Comparing the different models, it was obvious that the LR arrays had significantly larger heating power than the AP arrays, but the gap decreased after bone flap removal. What was found was that the increase in heating power was especially obvious after removing the bone flap in the insular lobe model, and the effect of the insulating layer on the heating power was different in each model.

## 4 DISCUSSION

In this study, we used a standard brain template, ICBM152, and intracranial lesions based on realistic head models derived from MRI data, to study the effect of skull defects on the intracranial electric field distribution and heating power in TTFields. We found that an increase in the intracranial electric field strength was accompanied by an increase in the heating power of gel in the presence of an incomplete skull. Skin dermatitis, the most common side effect of electric field therapy, is strongly correlated with heating power.^3^, ^4^, ^5^ We added an electric insulating layer to the skull defect region to reduce the heating power, allowing the increase in the heat production to be controlled when the bone flaps were removed. Moreover, the average electric field strength of the tumor shell and the heating power in each model were calculated by FEM.

Because there is no unified transducer array layout according to tumor location, size, and head parameters, and the object of this study was to investigate the effect of skull defects on the intracranial electric field distribution, the default array layout was adopted in each model in the study, and the calculation results only present the AP or the LR arrays, the pair of arrays that overlap with the location of the skull defect, since the effect of the skull defect on the other pair of arrays was almost negligible.

Several in vitro experiments have shown that the lower threshold of 1.0 V/cm is the minimum magnitude for cell division and thus inhibition of cell line proliferation. Moreover, the effect is positively correlated with electric field strength, with the inhibition effect reaching a maximum at 2.5 V/cm.^23^, ^24^ This modeling approach showed that the average field strength in the tumor shell increased in all models after removal of the bone flap. In addition, the extent of the increase varied; the average field strength in the tumor shell increased more in the frontal (49.1%) and temporal lobe models (49.6%) and less in the insular lobe (20.0%) and brain models (10.7%) (Figure 2). For all models, the average electric field strength of all the tumor shells exceeded the treatment threshold of 1 V/cm when the skull was intact. To a certain extent, this modeling approach also demonstrated the theoretical therapeutic effect of electric field therapy and the rationality of the default array layout.

Since the therapeutic efficacy of electric field therapy is dose‐dependent and the average field strength of the tumor shell reached 1 V/cm when the skull was intact, the ATV 1.0 (to reach the minimum treatment threshold) and ATV 2.5 (to reach the maximum treatment effect) had greater significance. For all models, an increase in ATV 1.0 was observed when skull defects, especially in the frontal lobe model (44.71%) and the brainstem (17.6%), showed a significant increase in ATV 1.0. In addition, almost all tumor shells reached 1 V/cm after removal of the bone flap. Therefore, the elevated ATV 1.0 in the frontal lobe and brainstem may be related to their lower basal ATV 1.0. In other words, the field strength of the AP arrays was smaller than that of the LR arrays and removing the bone flap may enhance the therapeutic effect of the AP arrays to some extent. Moreover, all the models other than the brainstem model showed an extremely significant increase in ATV 2.5. These results suggest that removing the bone flap can improve the field strength of TTFields during treatment, especially for superficial tumors in the frontal and temporal lobes. For the insular lobe and brainstem models, several previous studies have demonstrated that although specific arrays can improve the electric field strength in the tumor shell, the results are still not ideal compared to superficial tumors, and removing bone flap can also improve the electric field strength in the tumor shell.^10^ Further studies can be considered to improve the therapeutic effect of deep tumors in combination with a personalized array layout.

**FIGURE 4:**
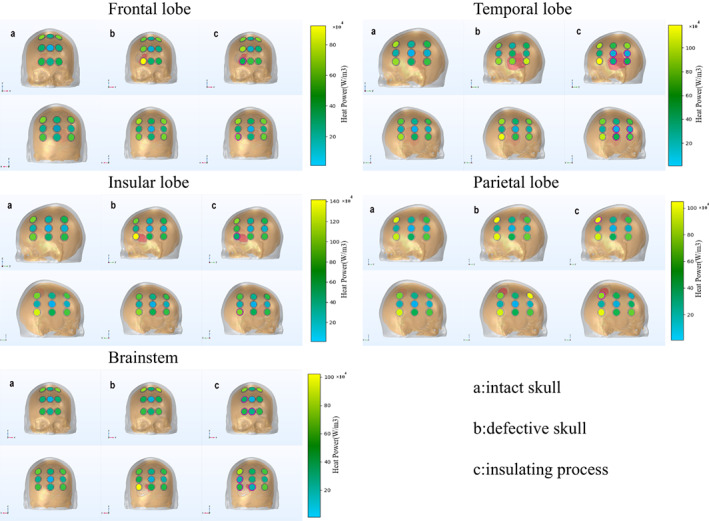
Array distribution and heating power of the gel for each model. The heatmap showed the heating power of each gel, and the electrode with the pink circle below the transducer meant that there was an insulating layer between the transducer and the hydrogel. Obvious enhancement of heating power was seen in the skull defect part. The gels with higher heating power tended to be located in the corner positions of the array, while the gels with lower heating power were often located in the center of the array. The red tissue was the tumor, the brown tissue was the skull, the gray tissue was the scalp, and the hole in the skull was the bone window.

Overall, Figure 4 showed that after removal of the bone flap, the maximum heating power increased in all models and there was a significant increase in the heating power of the gel at the site of the skull defect. On the other side of the arrays, interestingly, the numerical order of the heating power of the gel did not change in each case, and the heating power increased for individuals. What is more, the gel with higher heating power tended to be located in the corner positions of the array, while the gel with lower heating power was often located in the center of the array. This phenomenon was not mentioned in previous clinical data and would be verified in subsequent studies in the context of the actual patient situation. This meant that there was an edge effect not only on the electric field strength around the arrays, but also on the heating power of the gel of each array.^25^

It is known that in practical clinical applications, the temperature of the transducer is monitored, and the voltage will decrease when it reaches 41°C. During operation, the device can lower the voltage to make the heating power equal to the heat dissipation power, so that the temperature is controlled near 41°C. Since our results were based on the calculation of the default voltage, the actual tumor shell field strength needed to be further calculated based on the heating power. Therefore, we introduced a definition of “normalized electric field strength”, using the heating power of the intact skull case as a benchmark for the treatment unification of each case. In other words, it may represent the actual change in the electric field strength when the transducer with the highest heat power was adjusted to that value in the case of skull defect or insulating process.^26^

(3) $$E_{\text{normalized}}=\sqrt[{2}]{E_{2}\times \frac{W_{1}}{W_{2}}}$$

where $E_{\text{normalized}}$ denotes the normalized electric field strength, *E*_2_ indicates the average field strength in the skull defect or insulation process case, *W*_1_ is the maximum heating power for the skull intact case, and *W*_2_ is the maximum heating power for the skull defect or insulating process case.

In conclusion, Table 2 suggests that for the superficial frontal lobe model, the treatment outcome would be improved by removing the bone flap, and the insulating process can further improve the treatment effect. In the temporal and parietal lobe models, which contained superficial tumors, just removal of the bone flap was a better way to improve the therapeutic effect. With the insular lobe and brainstem models, a skull defect would result in a significant increase in heating power. In the insular lobe model, which contained a deeply located tumor with high electric field strength when the skull was intact, only removing the bone flap would slightly decrease the treatment effect. However, an insulating process at the base of skull defect would improve the treatment effect. In the brainstem model, which contained a deep‐located tumor with low electric field strength when the skull was intact, both the removal of bone flap and the insulating process were not effective. Concurrently, a phase II trial has been initiated to test the efficacy of skull remodeling surgery, although the method of removal of the bone flap is different, hopefully it is informative for the study.^16^ The innovative point, and clinical significance, of this study were to expand the indications for electric field therapy, but also to enhance the field strength of TTFields by certain active methods, and to demonstrate the existence of different treatment options for tumors in different locations. The insulating layer used in the study was a common material, and its physical parameters were similar to most polyethylene materials.

**TABLE 2:** Maximum heating power and normalized electric field strength of each model in each case.

	Heating power (W/m^3^)			$E_{\text{normalized}}$		
	Intact skull	Defective skull	Insulating process	Intact skull	Defective skull	Insulating process
Frontal lobe model	$7.26\times 10^{5}$	$9.25\times 10^{5}$	$7.43\times 10^{5}$	100%	132.1%	136.2%
Temporal lobe model	$9.91\times 10^{5}$	$1.07\times 10^{6}$	$1.19\times 10^{6}$	100%	138.3%	122.4%
Insular lobe model	$9.93\times 10^{5}$	$1.42\times 10^{6}$	$1.05\times 10^{6}$	100%	99.5%	108.2%
Parietal lobe model	$9.90\times 10^{5}$	$1.05\times 10^{6}$	$1.01\times 10^{6}$	100%	120.8%	113.2%
Brainstem model	$6.55\times 10^{5}$	$1.02\times 10^{6}$	$8.13\times 10^{5}$	100%	88.7%	96.5%

This study is a computational modeling study and the physical parameters of each tissue have a large impact on the accuracy of the study; every effort has been made to avoid this error by selecting parameters provided by authoritative organizations. Field correlation and anisotropic conductivity, which have an effect on the field distribution and efficacy, are not included in this study.^25^ In addition, some possible influencing factors, such as sweat, ambient temperature, and the patient's intradermal fat thickness, were not addressed in the study, and these factors may be further incorporated in subsequent studies. Due to the supporting role of the skull, the head shape may change after removal of the bone flap, which was not individualized to facilitate the calculation and placement of the transducer.

### AUTHOR CONTRIBUTION

Taian Jin and Zhangqi Dou were responsible for the study methods, data analysis, writing, and editing. Yu Zhao was responsible for the analysis. Boxing Wei, Biao Jiang, Jinghong Xu, Buyi Zhang, and Fei Dong were responsible for the clinical concepts and data acquisition. Chongran Sun and Jianmin Zhang were responsible for conceptualization, guidance, and supervision.

## CONFLICT OF INTEREST

None.

## ETHICS STATEMENT

The Second Affiliated Hospital, Zhejiang University School of Medicine, Center Institutional Review Board approved this modeling study using the de‐identified publicly available data. All patients provided informed consent.

## Data Availability

Output data from the models are available from Dr. Sun at sun.chongran@zju.edu.cn. Please contact Dr. Jin at 22018425@zju.edu.cn to collaborate on the use of the method for new analyses.
